# Successful treatment with HLA-matched peripheral hematopoietic stem cell transplantation for very severe hepatitis-associated aplastic anemia complicated with multidrug-resistant bacterial and fungal infections: A case report

**DOI:** 10.3389/fped.2022.828918

**Published:** 2022-10-31

**Authors:** Hua Li, Xiaofan Li, Xianling Chen, Nainong Li

**Affiliations:** ^1^ Hemopoietic Stem Cell Transplantation Center, Fujian Provincial Key Laboratory on Hematology, Clinical Research Center for Hematological Malignancies of Fujian Province, Fujian Institute of Hematology, Department of Hematology, Fujian Medical University Union Hospital, Fuzhou, China; ^2^ Department of Hematology, The Second Affiliated Hospital of Xiamen Medical College, Xiamen, China

**Keywords:** hematopoietic stem cell transplantation, severe infection, very severe anemia plastic, hepatitis-associated aplastic anemia, multidrug-resistant bacteria

## Abstract

Hepatitis-associated aplastic anemia (HAAA) is a life-threatening hematologic disorder characterized by bone marrow failure. Allogeneic hematopoietic stem cell transplantation (HSCT) is the first-line treatment for HAAA. Severe infection and complications in patients with very severe aplastic anemia are the challenges to the efficacy of HSCT. We report a rare case of successful transplantation with HLA-matched peripheral hematopoietic stem cells for a 15-year-old girl suffering from HAAA with multidrug-resistant bacterial and fungal infections. Through effectively controlling infection and optimal timing of transplantation by adjusting the conditioning regimen, the allo-HSCT was successfully performed for the patient. Updated data of following-up 26 months after transplantation showed that the patient was still in complete remission with a good quality of life. This case provided a reference for treating severely infected patients with HAAA before HSCT.

## Introduction

Hepatitis-associated aplastic anemia (HAAA) is rare and life-threatening aplastic anemia with obvious immune disorders. Bone marrow failure occurs within weeks to 1 year after the attack of acute hepatitis. It accounts for approximately 1%–5% of newly diagnosed acquired aplastic anemia (1). Patients usually have a high risk of early serious bacterial infection with severe neutropenia. The treatment efficacy for HAAA is poor. First-line treatment for HAAA is allogeneic stem cell transplantation from an HLA-matched sibling donor (MSD) (2). Five-year overall survival rates of hematopoietic stem cell transplantation (HSCT) for HAAA were 86.0% (3). Following the guideline for severe aplastic anemia (SAA), allogeneic MSD-HSCT is also applicable to HAAA (4). However, bacterial infections of multidrug-resistant (MDR) and invasive fungal infections (IFI) are life-threatening in patients with AA (5, 6), which is an obstacle to allogeneic HSCT. Therefore, the correct time of allogeneic stem cell transplantation for infected patients with SAA is extremely important. However, well-documented guidelines of HSCT for SAA patients with severe active infection are currently not available. Chinese expert consensus did not recommend HSCT for SAA patients with active infection (7). The British Society for Hematology and the European Society for Blood and Marrow Transplantation all recommended MSD-HSCT for young and adult patients with severe AA, but the best time of transplantation for patients with severe infection and hemorrhage is not clarified (8, 9). Therefore, whether HSCT should be performed for SAA with a severe infection and the optimal timing and strategy for HSCT needs to be investigated. Here, we report a case of successful treatment of MSD-HSCT for a 15-year-old girl with HAAA suffering from life-threatening MDR bacterial infection and IFI.

## Case representation

A 15-year-old Chinese girl suffering from fatigue, dizziness, and nausea was presented with severe bone marrow pancytopenia in October 2019. Acute jaundice hepatitis of unknown cause had been diagnosed 1 month before admission to our hospital. Complete blood count (CBC) showed that white blood cell counts were 0.19 × 10^9^/L, absolute neutrophil count (ANC) was 0.11 × 10^9^/L, hemoglobin level was 76 g/L, and platelet count was 12 × 10^9^/L. Blood exams showed that the transaminase and bilirubin were 20 times higher than the normal range (total bilirubin (TBIL) 251.7 μmol/L, direct bilirubin (DBIL) 179.3 μmol/L, indirect bilirubin (IBIL) 72.4 μmol/L, aspartate aminotransferase (AST) 1420 IU/L, alanine aminotransferase (ALT) 1757 IU/L). T-cell subsets of peripheral blood detected by flow cytometry showed that CD4^+^ was 9.11%, CD8^+^ was 69.83%, and CD4/CD8 rate was 0.14, which the ratio of CD4^+^/CD8^+^ reduced significantly. Bone marrow biopsy showed extremely hypocellular bone marrow (5%), reduced granulocytes and erythrocytes, hyperplasia of adipose components, no megakaryocytes, and an increased number of lymphocytes, plasma cells, and histiocytes. A cytogenetic study showed a normal karyotype. Serological tests for hepatitis (A, B, C), Epstein–Barr virus, cytomegalovirus (CMV), parvovirus B19, herpes, and HIV were negative. No positive genetic and molecular tests were found for congenital bone marrow failure diseases such as Fanconi anemia, Shwachman–Diamond, and dyskeratosis congenita. Paroxysmal nocturnal hemoglobinuria (PNH) diagnosis also was excluded. HAAA with negative viral serology was finally diagnosed.

**Figure 1:**
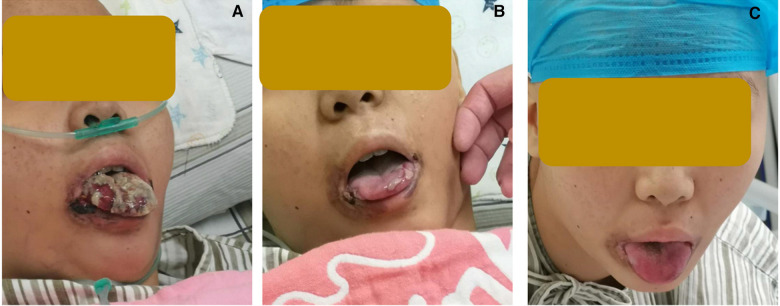
Change of oral fungal infections (**A**) on day −7 before HSCT, (**B**) on day +30 after HSCT, and (**C**) on day +55 after HSCT.

**Figure 2:**
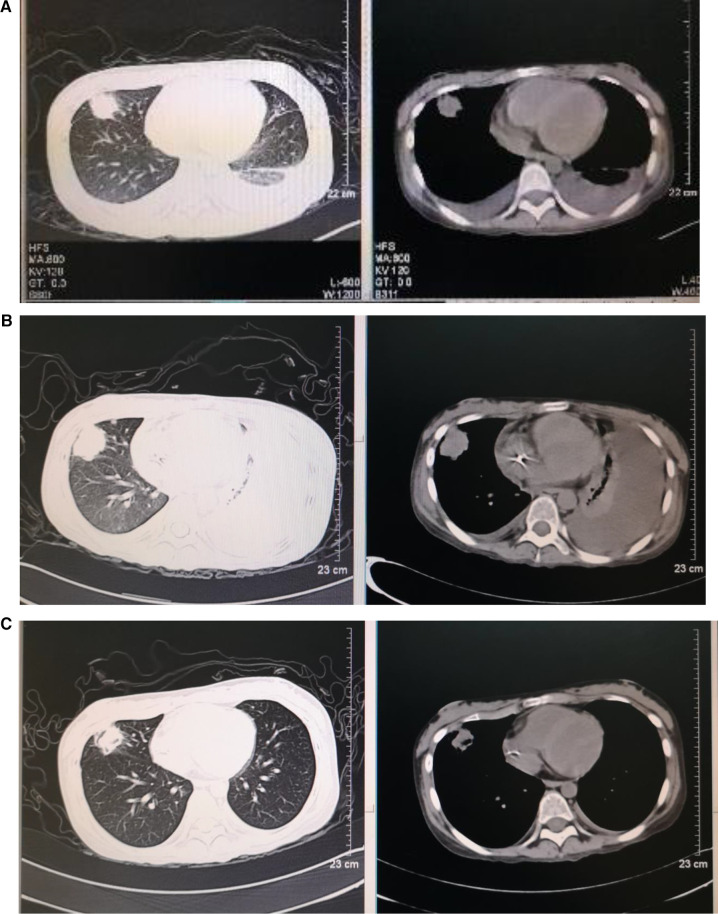
Dynamic images of pneumonia before and after transplantation (**A**–**C**). Chest high-resolution computed tomography: (**A**) 3.3 × 2.7 cm nodular shadow in the right upper lung, with bilateral pleural effusion on day −7 before HSCT, (**B**) 3.3 × 3.1 cm nodular shadow in the right upper lung, with bilateral pleural effusion and left atelectasis on day +22 after HSCT, and (**C**) 2.3 × 2.0 cm nodular shadow in the right upper lung on day +59 after HSCT.

**Figure 3:**
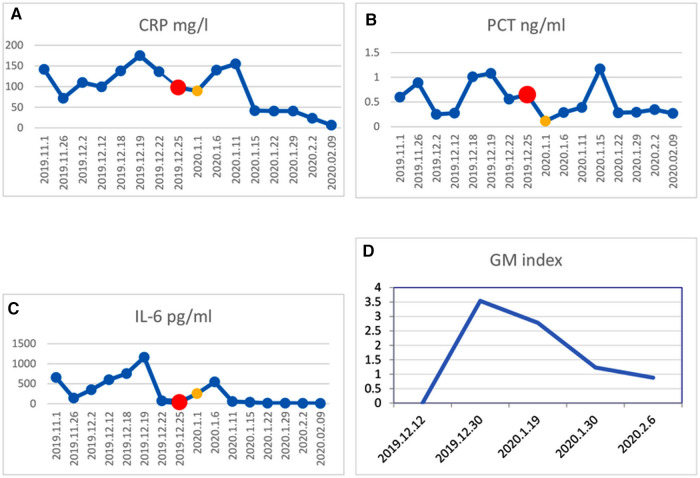
Change in inflammatory factor levels in CRP (**A**), PCT (**B**), IL-6 (**C**), and GM (**D**). The red dot in the line represents the beginning of preconditioning on Dec. 25, 2019, and the yellow dot represents the day of HSCT on Jan. 1, 2020. The patient received HSCT when inflammatory factors levels reduced markedly.

Treatments with liver protection, nutrition support, and immunosuppressive therapy (IST) with prednisone, cyclosporine (CsA), and hematopoiesis stimulation (granulocyte colony-stimulating factor (G-CSF), thrombopoietin receptor agonist (TPORA), and danazol) were started after admission. At the beginning of hospitalization, the patient refused antithymocyte globulin (ATG) or HSCT treatment due to the family’s financial distress. Due to pulmonary infection from agranulocytosis, β-lactamase inhibitors tazobactam (piperacillin–tazobactam, cefoperazone–sulbactam) and fluoroquinolones (levofloxacin) were used, successively. The patient’s liver function improved, and transaminases were back to normal after 1 month of liver protection treatment. However, the pancytopenia treatment showed no significant response. The patient remained platelet and red blood cell transfusion-dependent. Without effective infection control, the patient developed an oral fungal infection (Figure 1A) and pulmonary infections with MDR to bacteria and fungus due to long-term neutropenia and antibiotic use. An aspergilloma and a large number of pleural effusions were found by CT scanning (Figure 2A). Laboratory tests repeatedly showed sputum culture with *Pseudomonas aeruginosa* resistance to multidrug in December 2019. Serum (1,3)-β-D glucan (G-test) was negative, while galactomannan (GM-test) was positive. The bronchoalveolar lavage was rejected by the patient, and multiple fungal cultures from the sputum showed negative results. A conventional microdilution assay (CLSI methodology) showed the minimum inhibitory concentration values of imipenem and meropenem of 8 and >16, respectively. Empirical antibiotics Fosfomycin, Polymyxin, Meropenem, Voriconazole, and Micafungin could not control the patient’s infections. The patient’s symptom of fever, cough, and shortness of breath continued. Oral ulceration with fungal infection aggravated, and multidrug-resistant infection progressed to life-threatening sepsis. At the same time, the patient presented with vomiting blood and developed hemorrhagic shock. After effective antishock treatment, the patient’s condition was under control. With uncontrolled infections after multiple antibiotic treatments, the patient was given Ceftazidime Avibactam Sodium (2.5 g/kg i.v. q8h) and Fosfomycin (4 g b.i.d., i.v.) with a continuous infusion of Voriconazole and liposomal amphotericin B. At the same time, continuous drainage of the pleural fluid was performed. The patient’s status gradually improved, although the size of the fungus ball in the right upper lung showed no obvious shrinkage. The level of inflammatory markers of C-reactive protein (CRP) decreased from 175 to 68 mg/L, procalcitonin (PCT) from 3.5 to 0.1 ng/ml, and IL-6 from 1200 to 126 pg/ml (Figure 3). CT scan showed the bilateral pleural effusion was reduced significantly. The patient recovered from the hemorrhagic shock from gastrointestinal bleeding.

As the patient's health improved, we urgently considered hematopoietic stem cell transplantation with salvage therapy. When the patient family's financial situation improved, peripheral blood stem cells from her HLA-matched sister were transplanted. The conditioning regimen of peripheral blood stem cell transplantation was 2.5 mg/kg rabbit ATG (on day −5 to day −2) and 50 mg/kg/day cyclophosphamide (on days −5 to −2) from December 25 to 29 2019. Peripheral blood stem cells (CD34^+^ 10.15 × 10^6^/kg) were infused on day 0 (December 302019). CsA with the goal serum level in the range of 200–300 ng/ml was started on day −1. In total, 15 mg/m^2^/day MTX (day +1), 10 mg/m^2^/day MTX (+3, +6), Mycophenolate (day +7 to +45), and ruxolitinib (5 mg, bid) were administrated for graft versus host disease (GVHD) prophylaxis after neutrophil engraftment. MTX on day +11 was discontinued to reduce the risk of hematological toxicity. On day +15 post-transplantation, the patient’s ANC was 0.5 × 10^9^/L and it continued to increase. G-CSF was discontinued 3 days after ANC reached >1 × 10^9^/L. Platelet engraftment occurred on day +35. Then, the antibiotics were switched to antibacterial (cefoperazone/sulbactam, linezolid) and antifungal (caspofungin, liposomal amphotericin B) therapies. The patient’s temperature was returned to normal since day +18. The CT scan showed aspergilloma in the right upper lung with bilateral pleural effusion and left atelectasis on day +22, which seemed more serious than before HSCT. That might be because the patient suffered prolonged neutropenia before granulocyte engraftment (Figure 2B). With the patient’s health conditions improving rapidly, atelectasis and bilateral pleural effusion gradually disappeared. Shrank aspergilloma was seen on day +59 (Figure 2C). The patient’s CBC returned to a nearly normal range (WBC of 6.5 × 10^9^/L, Hb of 100 g/L, and platelets (PLT) of 104 × 10^9^/L) on day +72 with normal liver and kidney function (May 1 2020). Oral fungal infection was cured (Figure 1C).

After allo-HSCT, the patient’s condition continued to keep stable. Donor chimerism of 100% was achieved. Slightly chronic skin GVHD was observed and was well controlled with methylprednisolone. Following-up 26 months after transplantation, the patient was still in complete remission with a high-quality life. She was back to school after 1 year of allo-HSC transplantation.

## Discussion

HAAA is a rare form of AA that often occurs after an attack of acute hepatitis. Patients with HAAA develop severe pancytopenia weeks to months after an acute episode of self-relief hepatitis. The mean survival time after developing severe bone marrow aplasia is 2 months, and the fatality rate ranges from 78% to 88% (10). The treatment options for HAAA are IST and allogeneic HSCT. If a matched sibling donor is unavailable, IST would be the first-line treatment. The response rate of IST with ATG or ATG combined with CsA for HAAA is higher than 70% (1). IST regimen is associated with sustained remission for some HAAA children with hepatitis. It has been suggested to take earlier approaches for patients when HAAA is developing (11). HSCT is preferred over immune suppressive therapy if an HLA-identical donor is available (12). Mori et al. showed that allogeneic HSCT is safe with a low rate of transplant-related mortality (3). Our patient developed very severe aplastic anemia (VSAA) after an episode of seronegative acute hepatitis, although the patient’s liver function was improving. The patient’s transaminases (aspartate transaminase and alanine transaminase) were 20-fold higher than the normal range during the initial stages of the disease. The patient suffered MDR bacterial and invasive fungal infections, which were very difficult to be controlled. HSCT remains the only treatment option for the patient’s granulocyte recovery and infection control. However, the optimal management and consensus of HSCT for HAAA patients with severe infections was unavailable. The right timing of HSCT and appropriate transplant strategy for patients of HAAA having severe active infection remains to be investigated.

### Infection control

In this case, the patient suffered complicated conditions with life-threatening infections and gastrointestinal bleeding before stem cell transplantation and finally recovered from MSD-HSCT. HSCT is a relatively challenging treatment for patients with serious infections and complicated health situations. Infections caused by various MDR bacteria and fungi significantly increase the risk of death post-transplantation. Effective treatment choice for MDR *Pseudomonas aeruginosa* infection is extremely limited in patients with severe agranulocytosis, although polymyxins, antipseudomonal carbapenems, and antipseudomonal *β*-lactams are available (13, 14). Ceftazidime avibactam (CAZ-AVI) is a novel synthetic *β*-lactamase inhibitor for a range of MDR Gram-negative infections (15, 16), which was approved by the US FDA in 2017. CAZ-AVI was successfully used in patients with limited treatment options for complicated intra-abdominal infections and hospital-acquired, ventilator-associated pneumonia caused by carbapenem-resistant Enterobacteriaceae (CRE) and other multidrug Gram-negative bacteria (17, 18). In this case, the patient received initial antipseudomonal therapy combining polymyxins with carbapenems and other antibiotics combination treatment as well. The patient’s infection was still not controlled due to the MDR. After being treated with CAZ-AVI and Fosfomycin, the patient’s situation improved gradually. It was noted that invasive aspergillosis (IA) after HSCT is the most common IFI, with emerging cryptic species exhibiting resistance to commonly used antifungals, such as azoles. Liposomal amphotericin B showed efficacy in the second-line therapy of IA in adults and children (19). With the treatment of liposome amphotericin B and caspofungin for antifungals, the patient recovered gradually. The CT scan showed that the size of the aspergilloma reduced gradually.

### Timing of HSCT

The best timing of HSCT should be determined for patients, especially those with poor health status. Some studies showed that pre-existing invasive fungal infection and active bacterial infection were not contraindications for allogeneic HSCT (20, 21). In this case, we determined the timing of transplantation by patient’s temperature, inflammatory markers, and imaging status of infectious lesions. After controlling the infection with a temperature keeping around 38°C and having CRP, PCT, and IL-6 at acceptable levels, allo-HSCT was performed (Figure 2). CT scanning detected no new infection development after transplantation. Therefore, we would like to emphasize the important clinical information to decide the timing of HSCT for SAA patients with severe infection, which includes the following clinical markers: body temperature significantly decreased or tend to be normal, the levels of CRP, PCT, and other inflammatory factors declined moderately, no new or progressive infected lesions.

### Adjusted conditioning regimen

The condition guideline of HSCT for a patient with HAAA is not available. For having earlier stem cell engraftment to control progressing infection in the patient, we transplanted peripheral stem cells (not bone marrow) from her sister. Following the standard regimen of cyclophosphamide (Cy) plus ATG for young SAA patients with HLA-matched sibling donors, Cy (50 mg/kg, i.v., daily) and ATG (2.5 mg/kg, i.v., daily) were given from day −5 to −2. In total, 10.15 × 10^6^/kg CD34^+^ cells from her sister’s peripheral stem cells were transfused. For GVHD prophylaxis, we used CsA, a “short” course of MTX, MMF, and ruxolitinib to suppress the pathological immune reaction. To reduce the risk of hematological toxicity and promoting neutrophil engraftment, MTX was cut off on day +11. Due to discontinued MTX on day 11 and higher CD34^+^ cells, ruxolitinib was administered after neutrophil engraftment until day +100 to reduce the risk of GVHD. The patient’s neutrophils and platelets were grafted on day +15 and day +35, respectively, which was similar to other transplanted patients without severe infection. CMV infection was not activated with repeated CMV-DNA testing results (copy number <500). Grade-I GVHD was observed and manageable. Ruxolitinib is the first drug approved for the treatment of steroid-refractory acute graft versus host disease (aGVHD) (22), which played an important role in the avid treatment for aGVHD and cGVHD (23, 24). It was used for the patient because discontinued MTX and higher CD34^+^ cells might cause severe GVHD even in HLA-matched sibling HSCT. Therefore, the adjusted transplantation strategy we used could decrease the infection risk for HAAA patients receiving HSCT without negative effects on hematopoietic reconstitution and have better GVHD prevention.

## Conclusion

This is an uncommon case with VSAA, especially HAAA, having serious, complicated MDR bacterial and fungal infections before HSCT. Serious infections from long-term agranulocytosis are difficult to control. So, a timely selection and implementation of more effective treatment strategies for HAAA are highly recommended. Receiving MSD-HSCT as early as possible to avoid long-term agranulocytosis must be beneficial (8, 9). However, IST and HSCT were all delayed for this patient due to the patient’s poor economic conditions. With obstacles from serious active infection before HSCT, considering the factors of effectively controlling infection, the correct time point for HSCT and adjusted condition regimen are all very important for the patient. Finally, the patient achieved complete remission and experienced long-term survival. We hope that the experiences from this case would provide a reference for treating severely infected patients with HAAA before HSCT.

## Data Availability

The raw data supporting the conclusions of this article will be made available by the authors, without undue reservation.
